# Anagliptin ameliorates albuminuria and urinary liver-type fatty acid-binding protein excretion in patients with type 2 diabetes with nephropathy in a glucose-lowering-independent manner

**DOI:** 10.1136/bmjdrc-2017-000391

**Published:** 2017-07-07

**Authors:** Munehiro Kitada, Shin-ichi Tsuda, Kazunori Konishi, Ai Takeda-Watanabe, Mizue Fujii, Keizo Kanasaki, Makoto Nishizawa, Atsushi Nakagawa, Daisuke Koya

**Affiliations:** 1 Department of Diabetology and Endocrinology, Kanazawa Medical University, Ishikawa, Japan; 2 Division of Anticipatory Molecular Food Science and Technology, Medical Research Institute, Kanazawa Medical University, Uchinada, Ishikawa, Japan

**Keywords:** type 2 diabetes, nephropathy, anti-diabetic drugs, albuminuria

## Abstract

**Objective:**

The objective of this study is to elucidate the effect of anagliptin on glucose/lipid metabolism and renoprotection in patients with type 2 diabetic nephropathy.

**Methods:**

Twenty-five patients with type 2 diabetic nephropathy received anagliptin 200 mg/day for 24 weeks, and 20 patients who were switched to anagliptin from other dipeptidyl peptidase-4 (DPP-4) inhibitors were analyzed regarding primary and secondary endpoints. The primary endpoint was change in hemoglobin A1c (HbA1c) during treatment with anagliptin. Additionally, we evaluated changes in lipid data (low-density lipoprotein-cholesterol, high-density lipoprotein-cholesterol and triglyceride), blood pressure (BP), urinary albumin to creatinine ratio (UACR), liver-type fatty acid-binding protein to creatinine ratio (ULFABP) and renal function (estimated glomerular filtration rate and serum cystatin C) as secondary endpoints.

**Results:**

After switching to anagliptin from other DPP-4 inhibitors, the levels of HbA1c in the 20 participants showed no significant change, 7.5%±1.2% at 24 weeks compared with 7.3%±0.9% at baseline. The levels of the log10-transformed UACR were significantly reduced from 1.95±0.51 mg/g creatinine (Cr) at baseline to 1.76±0.53 mg/g Cr at 24 weeks after anagliptin treatment (p<0.01). The percentage change in the UACR (Δ%UACR) from baseline to 24 weeks was also significantly lower by −10.6% (p<0.001). Lipid data, systolic BP and renal function were not changed during anagliptin treatment. Additionally, ULFABP in eight participants, who had ≥5 µg/g Cr at baseline, was significantly decreased from baseline (8.5±2.8 µg/g Cr) to 24 weeks (3.1±1.7 µg/g Cr, p<0.01) after anagliptin treatment, and the percentage change in the ULFABP during anagliptin treatment was −58.1% (p<0.001).

**Conclusions:**

Anagliptin induced no significant change in HbA1c, lipid data, systolic BP and renal function. However, anagliptin reduced the UACR and ULFABP, although without a corresponding change in HbA1c, indicating direct action of anagliptin on renoprotection in patients with type 2 diabetic nephropathy.

**Significance of the study**

**What is already known about this subject?**

- Previous clinical reports already exhibit that dipeptidyl peptidase-4 (DPP-4) inhibitors ameliorate diabetic nephropathy, such as albuminuria, in a glucose-lowering effect.

**What are the new findings?**

- Our current study demonstrates that administration of anagliptin, which is switched from other DPP-4 inhibitors, in patients with type 2 diabetes with nephropathy showed that the levels of urinary albumin to creatinine ratio were significantly reduced after 24 weeks. However, the levels of hemoglobin A1c in patients showed no significant change during the treatment.
- We also found that treatment with anagliptin significantly decreased urinary liver-type  fatty acid-binding protein excretion after 24 weeks.

**How might these results change the focus of research or clinical practice?**

- Anagliptin may exert beneficial effects for renoprotection in patients with type 2 diabetes with nephropathy in a glucose-lowering-independent manner.

## Introduction

The prevalence of diabetes mellitus has been increasing worldwide in recent years. Long-term diabetes results in vascular changes and dysfunction, and its complications are the major causes of morbidity and mortality in patients. Among diabetic vascular complications, nephropathy is recognized as a leading cause of end-stage renal disease (ESRD) and an independent risk factor for cardiovascular diseases (CVD).^1^ The early clinical sign of diabetic nephropathy is elevated urinary albumin excretion, referred to as microalbuminuria, which progresses to overt proteinuria. Microalbuminuria in patients with diabetes has been recognized as a useful biomarker for diagnosing diabetic nephropathy and as a predictive factor for progression to ESRD.^2^ Additionally, microalbuminuria has been shown to be closely associated with an increased risk of cardiovascular morbidity and mortality.^3–5^ Therefore, microalbuminuria is a biomarker for the diagnosis of diabetic nephropathy and an important therapeutic target for improving the prognosis of renal and cardiovascular risk in patients with diabetes.^6^ Previous clinical data also showed that urinary liver-type free fatty acid-binding protein (L-FABP), which is associated with renal tubulointerstitial damage and oxidative stress, may be a predictive marker for renal and cardiovascular prognosis in patients with type 2 diabetes.^7 8^

Multifactorial management, including diet therapy and glycemic, blood pressure (BP) and lipid control, is recommended for diabetic nephropathy.^2 9 10^ Among the multifactorial treatments, intensive glycemic control in type 2 diabetes significantly reduced diabetes-induced microvascular events, mainly as a consequence of a reduction in nephropathy.^2^ However, intensive glycemic control, accompanied by hypoglycemia, is closely related to increased mortality, which is associated with increased incidence of CVD.^11 12^ Therefore, avoiding hypoglycemia is important in the treatment of patients with diabetes, in particular those who have diabetic nephropathy, because they are a high-risk group for CVD. Treatment with dipeptidyl peptidase-4 (DPP-4) inhibitors, which are oral antidiabetic agents, results in improvements in the blood glucose levels in patients with diabetes following stimulation of endogenous insulin secretion, inhibition of glucagon release and reduction of gastric emptying via the enhanced production of incretin hormones. DPP-4 inhibitors enhance active levels of  glucagon-like peptide-1 (GLP-1) and gastric inhibitory polypeptide (GIP) via inhibition of cleaving and inactivating these incretins by DPP-4 enzyme. DPP-4 inhibitors have become widely accepted in clinical practice because of their low risk of hypoglycemia. In addition to the glucose-lowering effect, previous data from animal and clinical studies demonstrate that DPP-4 inhibitors, including sitagliptin,^13–18^ linagliptin,^19–23^ alogliptin,^24^ vildagliptin^25 26^ or saxagliptin,^27^ have pleiotropic beneficial effects such as renoprotection or antiatherogenesis, which are independent of the glucose-lowering effect. Additionally, anagliptin shows serum lipid-lowering effects, which have not yet been observed with the other DPP-4 inhibitors. However, there are not sufficient clinical data regarding the renoprotective effect of anagliptin in patients with diabetes. Therefore, the aim of this study is to investigate the possible effects of anagliptin on glycemic/lipid control and renal function, including albuminuria, in patients with type 2 diabetes with nephropathy.

## Research design and method

### Subjects

A total of 48 participants with type 2 diabetes (30 men and 18 women) were selected for the present study from outpatients who visited the Department of Endocrinology and Metabolism at Kanazawa Medical University Hospital. The entry criteria included (1) age ≥20 years old, (2) type 2 diabetes with hemoglobin A1c (HbA1c) ≥6.0%, (3) urinary albumin to creatinine (Cr) ratio (UACR) ≥30 mg/g Cr in spot urine for screening of diabetic nephropathy, and (4) treatment with diet, exercise therapy and oral antidiabetic agents (glimepiride ≤2 mg/day or gliclazide ≤40 mg/day or glibenclamide ≤1.25 mg/day). The exclusion criteria were (1) type 1 diabetes, (2) treatment with insulin therapy, (3) severe diabetic metabolic complications such as ketoacidosis, (4) severe liver dysfunction, (5) hemodialysis, (6) severe chronic heart failure, (7) pregnant or nursing women and those who might be pregnant, and (8) any patient whom the investigator judged to be inappropriate for this study. Patients were given detailed explanations of the study protocol. Informed consent was obtained from each patient. The study protocol was approved by the Ethical Committee of Kanazawa Medical University. The trial was registered with the University Hospital Medical Information Network (UMIN No 000012802).

### Study protocol

The present study was an open-label, prospective study. At the start of the study, anagliptin 200 mg/day was added to other oral antidiabetic agents such as sulfonylurea (SU), metformin, an α-glucosidase inhibitor (α-GI), pioglitazone and a sodium-glucose cotransporter 2 (SGLT2) inhibitor or, when participants received other DPP-4 inhibitors, the DPP-4 inhibitor was switched to anagliptin 200 mg/day. In addition, in some cases, the anagliptin dose was increased to up to 400 mg/day after 12 weeks, if the physician judged glucose control in the patients to be insufficient. Participants were assessed for the following parameters before the start of the study, 12 and 24 weeks after the addition of anagliptin or when switching to anagliptin: No changes were made to the type and dose of glucose-lowering agents, renin–angiotensin system (RAS) inhibitors such as ACE inhibitors (ACEIs), angiotensin receptor II blockers (ARBs) or spironolactone during the study period. These agents had been prescribed for at least 3 months before the study.

After performing a screening of UACR ≥30 mg/g Cr in spot urine, diabetic nephropathy was finally diagnosed by an UACR ≥30 mg/g Cr in the first urine in the early morning. The primary endpoint of the study was change in HbA1c during the treatment with anagliptin. Additionally, we evaluated the changes in lipid data (low-density lipoprotein-cholesterol (LDL-C), high-density lipoprotein-cholesterol (HDL-C) and triglyceride (TG) levels), UACR and urinary L-FABP excretion as secondary endpoints.

### Measurements

Blood samples were collected in the morning after an overnight fast. First urine in the early morning sample was collected at the home of participants, and urine was carried in a cooling box to the hospital. HbA1c was measured using an automated analyzer, HLC-723 G11 (TOSHO, Tokyo, Japan). Serum LDL-C and HDL-C levels were measured using enzymatic methods (Qualigent HDL-C and Qualigent LDL-C, Sekisui Medical, Tokyo, Japan). Serum TG levels were measured using enzymatic assays (Kyowa Medex, Tokyo, Japan). Urinary albumin was measured by immunonephelometry using a kit from NITTOBO MEDICAL (Tokyo, Japan). Because of their skewed distribution, the UACR data were log10-transformed before analysis. The results of the analysis were back-transformed to obtain geometric means of the UACR of the 24-week value to the baseline value; the values then were expressed as percentage change in the adjusted geometric mean of the UACR ratios of the 24-week value to the baseline value. Serum and urinary Cr were measured using enzymatic assays (Sekisui Medical), and the estimated glomerular filtration rate (eGFR) was calculated as 194×serum creatinine^−1.094^×age^−0.287^ in men and as 194×serum creatinine^−1.094^×age^−0.287^×0.739 in women.^28^ Urinary L-FABP was measured by a chemiluminescent enzyme immunoassay, using a Lumipulse L-FABP assay (Fujirebio, Tokyo, Japan), and urinary L-FABP excretion was expressed as the urinary L-FABP to Cr ratio. Serum cystatin C was measured by Latex immunoturbidimetric methods (LSI Medience, Tokyo, Japan).

### Statistical analysis

Statistical analyses were performed with a StatView V.5 system (Abacus Concepts, Berkeley, California, USA) for Windows. All values are summarized as the mean and SD unless otherwise indicated. Differences in the percentage change in the UACR and urinary L-FABP before and after administration of anagliptin were assessed by a paired t-test. A Wilcoxon signed-rank test was performed as appropriate for comparison of the two groups. The unpaired t-test was performed as comparison of the two groups on HbA1c, lipid data, BP, body mass index (BMI) and renal function. The correlation of two variables was analyzed by single linear regression analysis. Statistical significance was defined as p<0.05.

## Results

**Figure 1:**
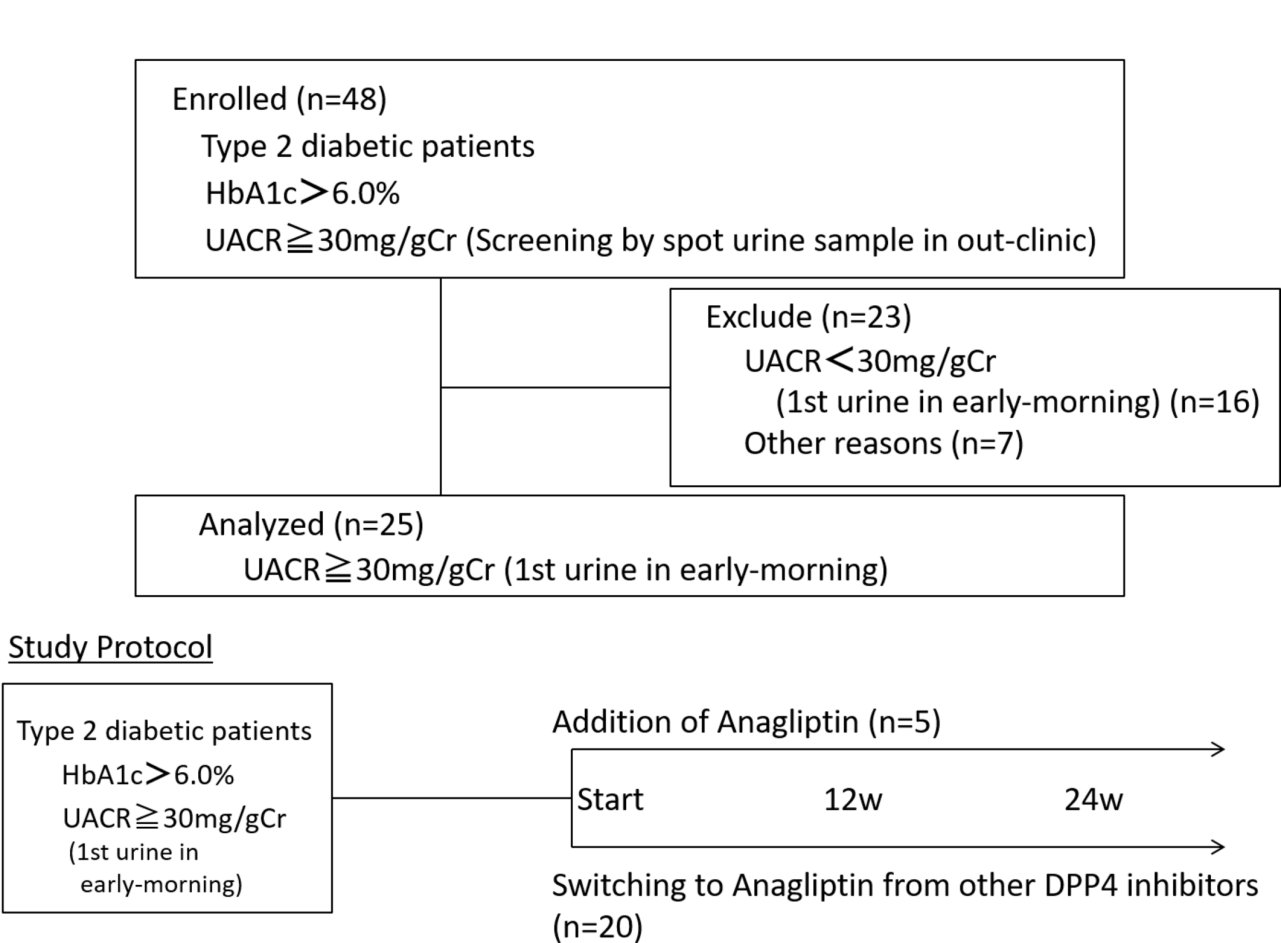
Patient disposition and study protocol. The 25 participants showed 30 mg/g creatinine (Cr) in the urinary albumin to Cr ratio (UACR) in the first urine in the early morning and were diagnosed with diabetic nephropathy. They received anagliptin as an additional treatment (n=5) or were switched from other dipeptidyl peptidase-4 (DPP-4) inhibitors (n=20), and were evaluated at the start of the study and after 12 and 24 weeks. HbA1c, hemoglobin A1c.

Patient disposition is shown in figure 1. Initially, 48 subjects who exhibited albuminuria of more than 30 mg/g Cr in spot urine were enrolled in this study. However, seven subjects were excluded for several reasons, which were diarrhea (n=2), elevation of BP (n=1), colon diverticulitis (n=1), worsening of depression (n=1), increased drowsiness (n=1) and withdrawal of consent before the beginning of treatment with anagliptin (n=1). Furthermore, 16 subjects were also excluded because their albuminuria was less than 30 mg/g Cr in the early-morning first urine. Therefore, we evaluated 25 subjects for the analysis of diabetic nephropathy in this study. Of 25 subjects, 20 subjects were switched to anagliptin from other DPP-4 inhibitors, and in 5 subjects anagliptin was additionally administered.

Baseline clinical and biochemical characteristics in two groups (shown as a group of switch (n=20) and a group of addition (n=5)), as well as concomitant background therapies, are shown in tables 1 and 2. The mean age was 67.6±9.0 and 71.2±4.6 years old, men:women=15:5 and 3:2, baseline BMI of the study population was 25.1±4.0 kg/m^2^ and 26.6±3.3 kg/m^2^, and the duration of diabetes was 15.1±7.6 and 12.4±4.8 years, respectively, in the two groups. Baseline HbA1c levels were 7.3%±0.9% and 7.8%±0.9%, and fasting glucose levels were 153.7±38.5 mg/dL and 182.8±80.5 mg/dL. Lipid data were 91.5±25.4 mg/dL and 104.2±17.6 mg/dL for LDL-C, 50.2±13.9 mg/dL and 46.8±11.0 mg/dL for HDL-C, and 165.6±98.7 mg/dL and 123.2±46.6 mg/dL for TG, respectively. Alanine aminotransferase levels were 20.3±9.8 IU/L and 21.0±6.4 IU/L, and uric acid levels were 5.5±1.3 mg/dL and 4.6±1.3 mg/dL, respectively. The median eGFR and serum cystatin C at baseline were 74.0±18.4 mL/min/1.73 m^2^ and 66.3±23.9 mL/min/1.73 m^2^, and 0.95±0.23 mg/dL and 0.98±0.35 mg/dL, and the UACR values at baseline were 206.4±343.9 mg/g Cr and 172.3±139.4 mg/g Cr, respectively. We assessed urinary L-FABP excretion in the participants showing more than 5 µg/g Cr at baseline, and the median urinary L-FABP excretion was 8.5±2.8 and 6.4±0.95 µg/g Cr, respectively. At entry of study, in each of the two groups, 80% of the participants had microalbuminuria, and macroalbuminuria was noted in 20% of the individuals. All participants received oral antidiabetic agents including SU (55% and 40%), metformin (75% and 100%), an α-GI (20% and 0%), pioglitazone (10% and 0%) and an SGLT2 inhibitor (5% and 0%) at baseline. Twenty participants received a DPP-4 inhibitor, which includes sitagliptin (n=7, 35%), teneligliptin (n=5, 25%), alogliptin (n=3, 15%), vildagliptin (n=3, 15%) and linagliptin (n=2, 10%). Fifteen participants (75%) in the group of switch (n=20) received antihypertensive therapy at baseline. Of the 15 participants, 13 participants (87%) received RAS inhibitors such as ACEIs (n=4), ARBs (n=9) or spironolactone (n=2) at baseline, with two participants receiving dual RAS blockade, using ACEI and ARBs, or ARBs and spironolactone. Fourteen participants (70%) in the group of switch were treated with lipid-lowering therapy. Of the 14 participants, 12 participants received statins (85%).

**Table 1:** Baseline clinical and biochemical characteristics

n	20 (Switch)	5 (Addition)
Male:female	15:5	3:2
Age (years)	67.6±9.0	71.2±4.6
BMI (kg/m^2^)	25.1±4.0	26.6±3.3
BP (mm Hg)	130.9±12.3/71.8±10.8	145.4±18.5/73.0±6.8
HbA1c (%)	7.3±0.9	7.8±0.9
FPG (mg/dL)	153.7±38.5	182.8±80.5
LDL-C (mg/dL)	91.5±25.4	104.2±17.6
HDL-C (mg/dL)	50.2±13.9	46.8±11.0
TG (mg/dL)	165.6±98.7	123.2±46.6
ALT (IU/L)	20.3±9.8	21.0±6.4
UA (mg/dL)	5.5±1.3	4.6±1.3
eGFR (mL/min/1.73 m^2^)	74.0±18.4	66.3±23.9
eGFR>90(mL/min/1.73 m^2^), n (%)	5 (25)	1 (20)
eGFR 60–90 (mL/min/1.73 m^2^), n (%)	10 (50)	2 (40)
eGFR 30–60 (mL/min/1.73 m^2^), n (%)	5 (25)	2 (40)
Cystatin C (mg/dL)	0.95±0.23	0.98±0.35
UACR (mg/g Cr)	206.4±343.9	172.3±139.4
UACR 30–300 (mg/g Cr), n (%)	16 (80)	4 (80)
UACR>300 (mg/g Cr), n (%)	4 (20)	1 (20)
UACR (log) (mg/g Cr)	1.95±0.51	1.99±0.38
ULFABP>5.0 (μg/g Cr), n (%)	8 (40)	3 (60)
ULFABP (μg/g Cr)	8.5±2.8	6.4±0.95
Duration of diabetes (years)	15.1±7.6	12.4±4.8

Data are the mean±SD, or n (%).

ALT, alanine transaminase; BMI, body mass index; BP, blood pressure; Cr, creatinine; eGFR, estimated glomerular filtration rate; FPG, fasting plasma glucose; Hb1Ac, hemoglobin A1c; HDL-C, high-density lipoprotein-cholesterol; LDL-C, low-density lipoprotein-cholesterol; TG, triglyceride; UA, uric acid; UACR, urinary albumin to creatinine ratio; ULFABP, urinary liver-type fatty acid-binding protein to creatinine ratio.

**Table 2:** Baseline background therapies for diabetes, hypertension and dyslipidemia

Antidiabetic background therapy at baseline, n (%)	20 (100) (Switch)	5 (100) (Addition)
Metformin, n (%)	15 (75)	5 (100)
SUs, n (%)	11 (55)	2 (40)
DPP-4 inhibitors, n (%)	20 (100)	–
Sitagliptin, n (%)	7 (35)	–
Teneligliptin, n (%)	5 (25)	–
Alogliptin, n (%)	3 (15)	–
Vildagliptin, n (%)	3 (15)	–
Linagliptin, n (%)	2 (10)	–
α-GIs, n (%)	4 (20)	0
Pioglitazone, n (%)	2 (10)	0
SGLT2 inhibitors, n (%)	1 (5)	0

**Table:** 

Antihypertensive background therapy at baseline, n (%)	15 (75)	3 (60)
RAS inhibitors, n (%)	13 (87)	2 (67)
ACEIs, n	4	0
ARBs, n	9	2
ACEI+ARB, n	1	0
Spironolactone, n	2	0
Ca antagonists, n (%)	11 (73)	3 (60)
Diuretics, n (%)	0 (0)	1 (20)
β blockers, n (%)	1 (7)	1 (20)
α-Methyldopa, n (%)	2 (13)	0

**Table:** 

Lipid-lowering background therapy, n (%)	14 (70)	2 (40)
Statins, n (%)	12 (85)	2 (40)
Fibrate, n (%)	2 (14)	0
Ezetimibe, n (%)	1 (0.7)	0

α-GI, α-glucosidase inhibitor; ACEI, ACE inhibitor; ARB, angiotensin II receptor blocker; DPP-4, dipeptidyl peptidase-4; RAS, renin–angiotensin system; SGLT2, sodium-glucose cotransporter 2; SU, sulfonylurea.

In a group with additional treatment with anagliptin (n=5, including one participant with anagliptin dose of up to 400 mg/day after 12 weeks), HbA1c was significantly decreased at 12 and 24 weeks (6.9%±0.5%, p<0.01 and 6.7%±0.3%, p<0.05) from baseline (7.8%±0.9%), respectively (see online supplementary figure 1A). The UACR from baseline to 12 or 24 weeks was not significantly decreased in this group, but there was a tendency toward reduction (see online supplementary figure 1B,C). However, a single linear regression analysis between Δ%UACR and ΔHbA1c at 24 weeks showed significant correlation (r=0.904, p=0.035) (see online supplementary figure 1D). However, since the number of participants who received additional treatment with anagliptin was small (n=5) in this study, limited conclusions can be drawn. Therefore, we excluded five patients who received additional treatment with anagliptin, and analyzed 20 subjects who were switched to anagliptin from other DPP-4 inhibitors for evaluating the effect of anagliptin on glycemic control, lipid data and diabetic nephropathy in this study.

10.1136/bmjdrc-2017-000391.supp1 Supplementary data 1

**Figure 2:**
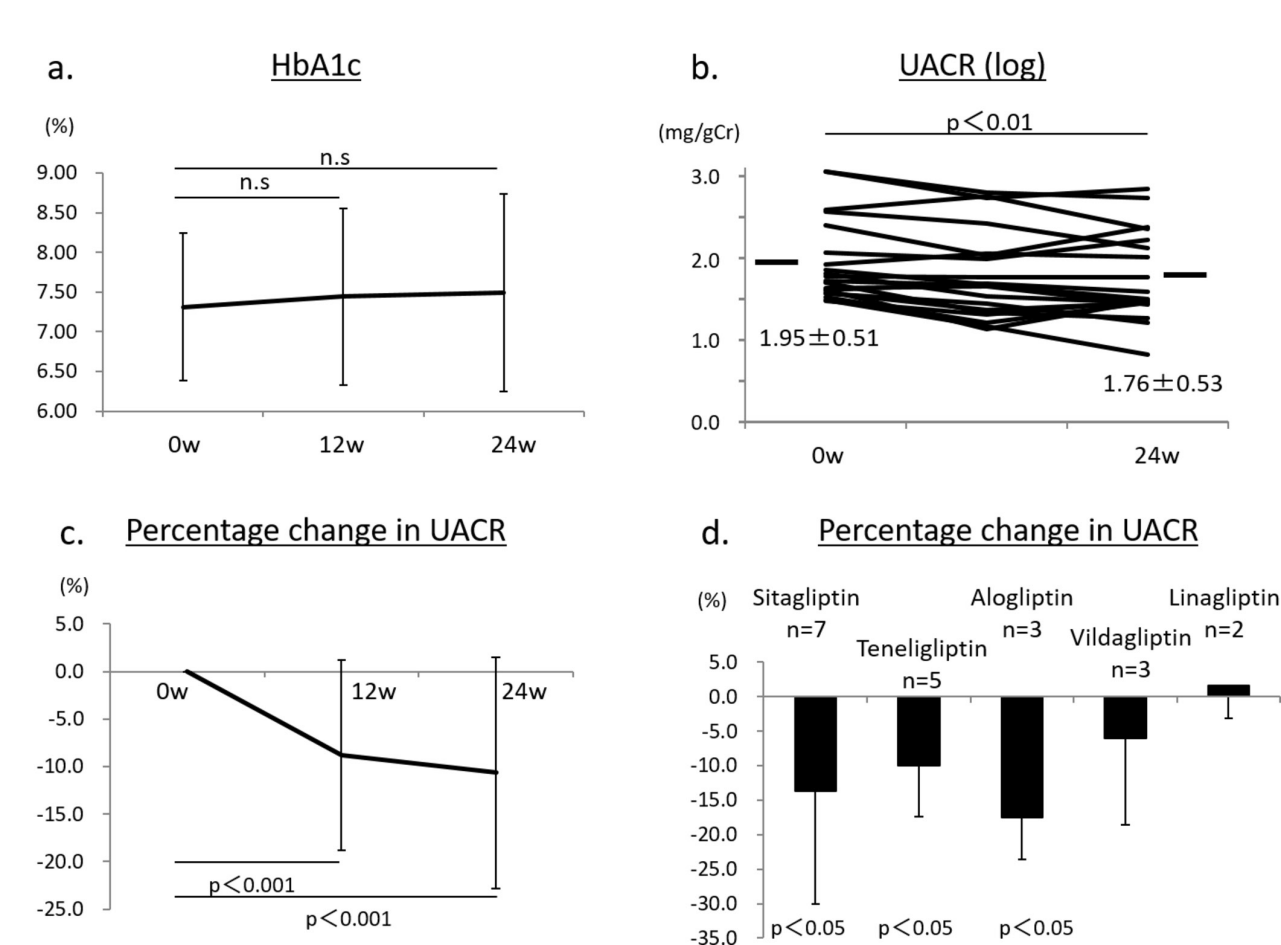
(A) Hemoglobin A1c (HbA1c) values at baseline and after treatment with anagliptin in 20 participants at 12 and 24 weeks.  (B) Urinary albumin to creatinine ratio (UACR) (log) values in 20 participants at baseline and after treatment with anagliptin at 24 weeks. p<0.01 versus baseline. (C) Percentage change in the UACR in 20 participants from baseline to after treatment with anagliptin at 12 and 24 weeks. p<0.001 versus baseline. (D) Change in the UACR after switching from sitagliptin, alogliptin, vildagliptin, teneligliptin or linagliptin to anagliptin. p<0.05 versus baseline. Error bars represent SD. n.s, denotes not significant.

After treatment with anagliptin switching from other DPP-4 inhibitors (n=20), HbA1c was not significantly changed, 7.4%±1.1% at 12 weeks and 7.5%±1.2% at 24 weeks, compared with baseline HbA1c (7.3%±0.9%) (figure 2A). The UACR (log) was significantly reduced after 24 weeks of treatment with anagliptin (1.76±0.53 mg/g Cr, p<0.01), compared with that at baseline (1.95±0.51 mg/g Cr) (figure 2B). The percentage change in UACR (Δ%UACR) from baseline to 12 or 24 weeks was also significantly lower by −8.8% at 12 weeks (p<0.001) and by −10.6% at 24 weeks (p<0.001) (figure 2C). The significant reduction in the UACR induced by anagliptin might be due to switching from sitagliptin, alogliptin and teneligliptin. By contrast, switching from vildagliptin and linagliptin to anagliptin seemed to show no effect on urinary albumin excretion (figure 2D). Lipid data including LDL-C, HDL-C and TG were not changed during treatment with anagliptin (figure 3A–C). We also found no significant change in systolic BP during treatment with anagliptin (figure 3D), and the reduction of Δ%UACR from baseline to 12 or 24 weeks was independent of receiving RAS inhibitors (figure 3E,F). BMI showed no significant change after 12 and 24 weeks of treatment with anagliptin compared with that at baseline (figure 3G). Renal function, which was evaluated by measurement of eGFR and serum cystatin C levels, showed no significant change during anagliptin treatment (figure 3H,I). Furthermore, we assessed urinary L-FABP excretion. We analyzed eight participants who had more than 5 µg/g Cr of urinary L-FABP excretion at baseline in switching to anagliptin treatment group. Urinary L-FABP excretion was significantly decreased from baseline (8.5±2.8 µg/g Cr) to 24 weeks (3.1±1.7 µg/g Cr, p<0.01) after treatment with anagliptin (figure 4A), and the percentage change in urinary L-FABP excretion (Δ%ULFABP (L-FABP to creatinine ratio)) during treatment with anagliptin was −58.1% (p<0.001) (figure 4B).

**Figure 3:**
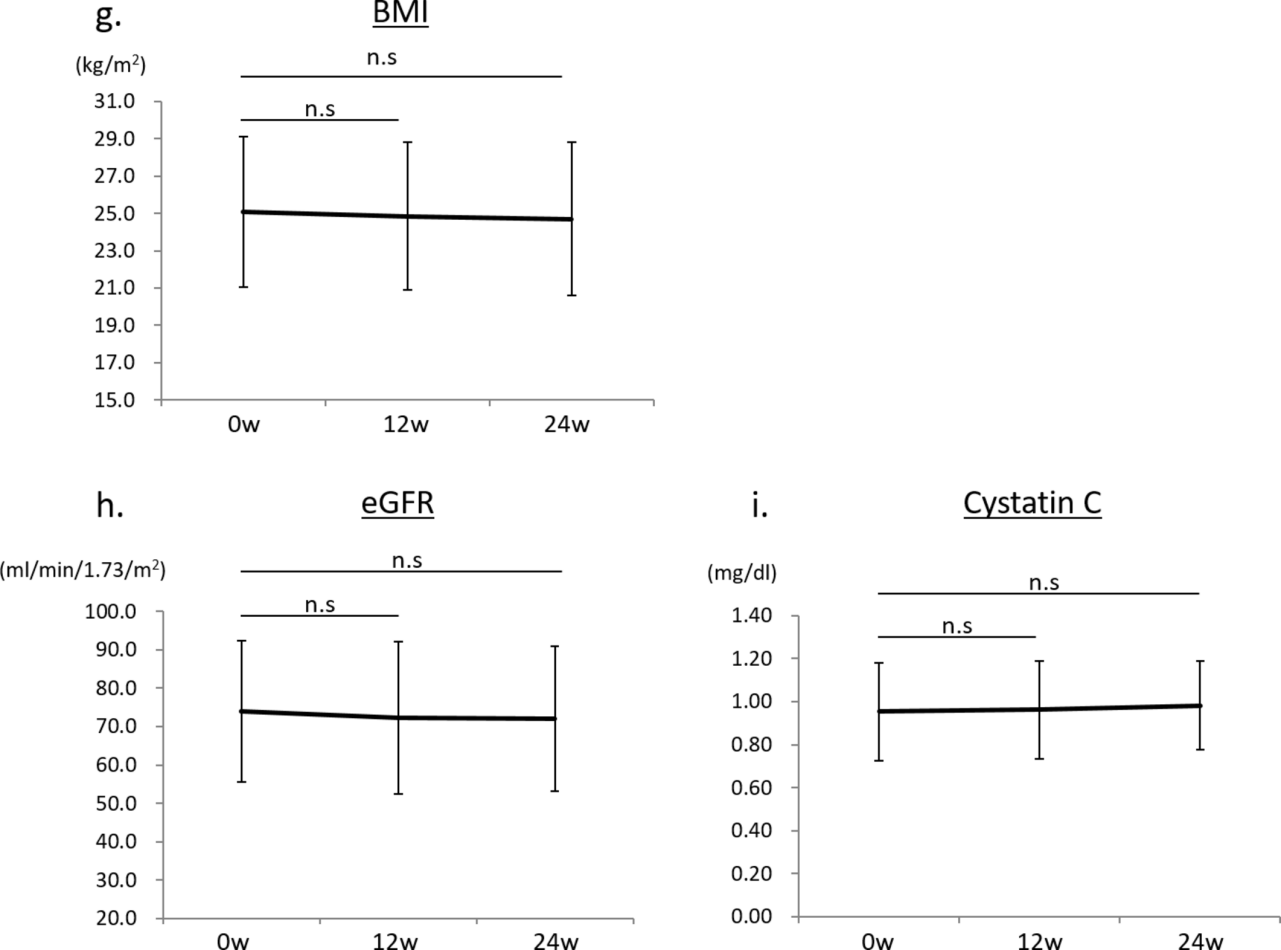
(A) Low-density lipoprotein-cholesterol (LDL-C) values at baseline and after treatment with anagliptin in 20 participants at 12 and 24 weeks. (B) High-density lipoprotein-cholesterol (HDL-C) values at baseline and after treatment with anagliptin in 20 participants at 12 and 24 weeks. (C) Triglyceride (TG) values at baseline and after treatment with anagliptin in 20 participants at 12 and 24 weeks. Error bars represent SD. n.s denotes not significant.

**Figure 3:**
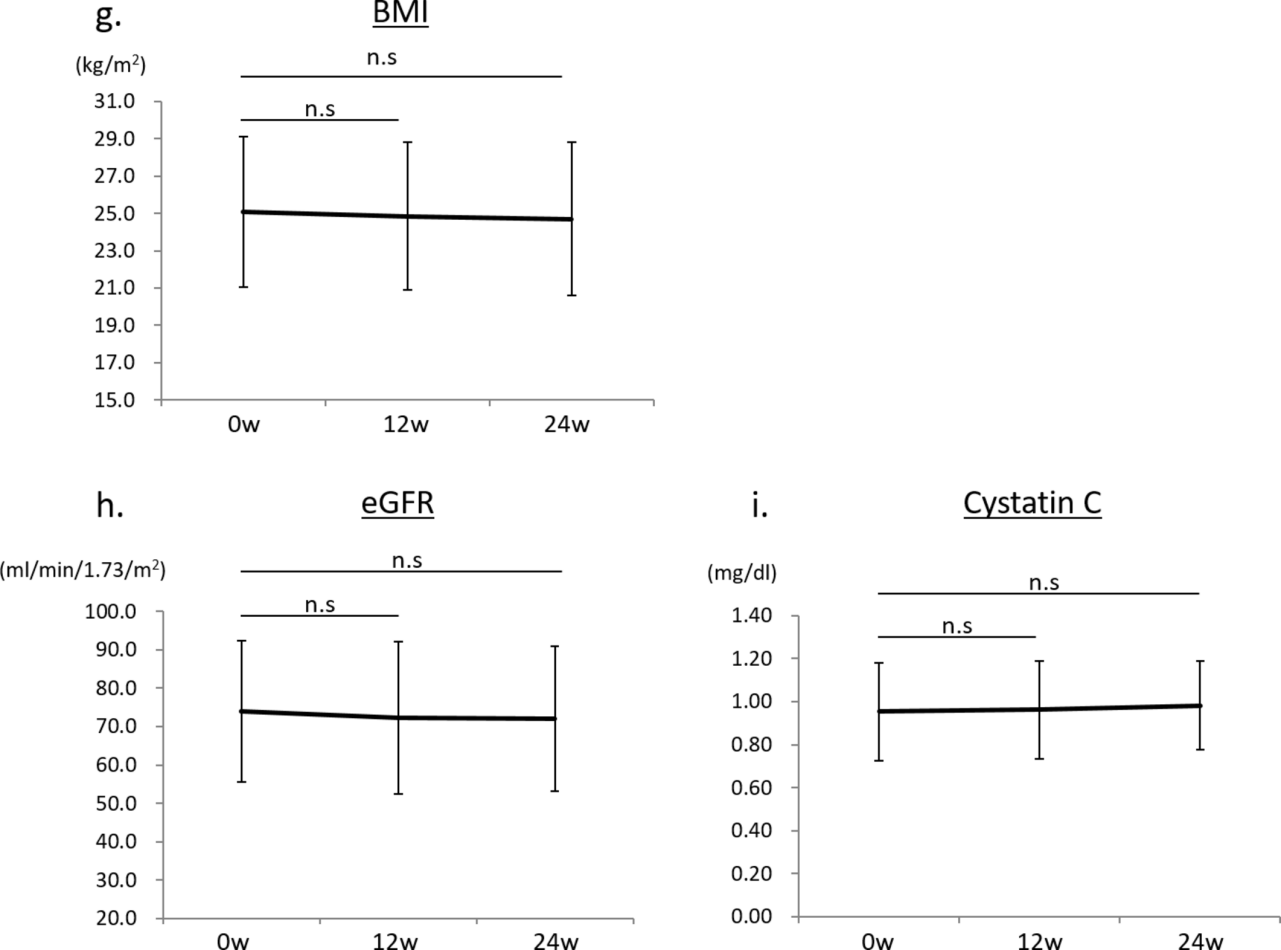
(D) Systolic blood pressure (BP) values at baseline and after treatment with anagliptin in 20 participants at 12 and 24 weeks. . (E) Percentage change in the urinary albumin to creatinine ratio (UACR) in 13 participants who received renin–angiotensin system (RAS) inhibitors from baseline to after treatment with anagliptin at 12 and 24 weeks. p<0.05 versus baseline. (F) Percentage change in the UACR in 7 participants who did not receive RAS inhibitors from baseline to after treatment with anagliptin at 12 and 24 weeks. p<0.05 versus baseline. Error bars represent SD. n.s denotes not significant.

**Figure 3:**
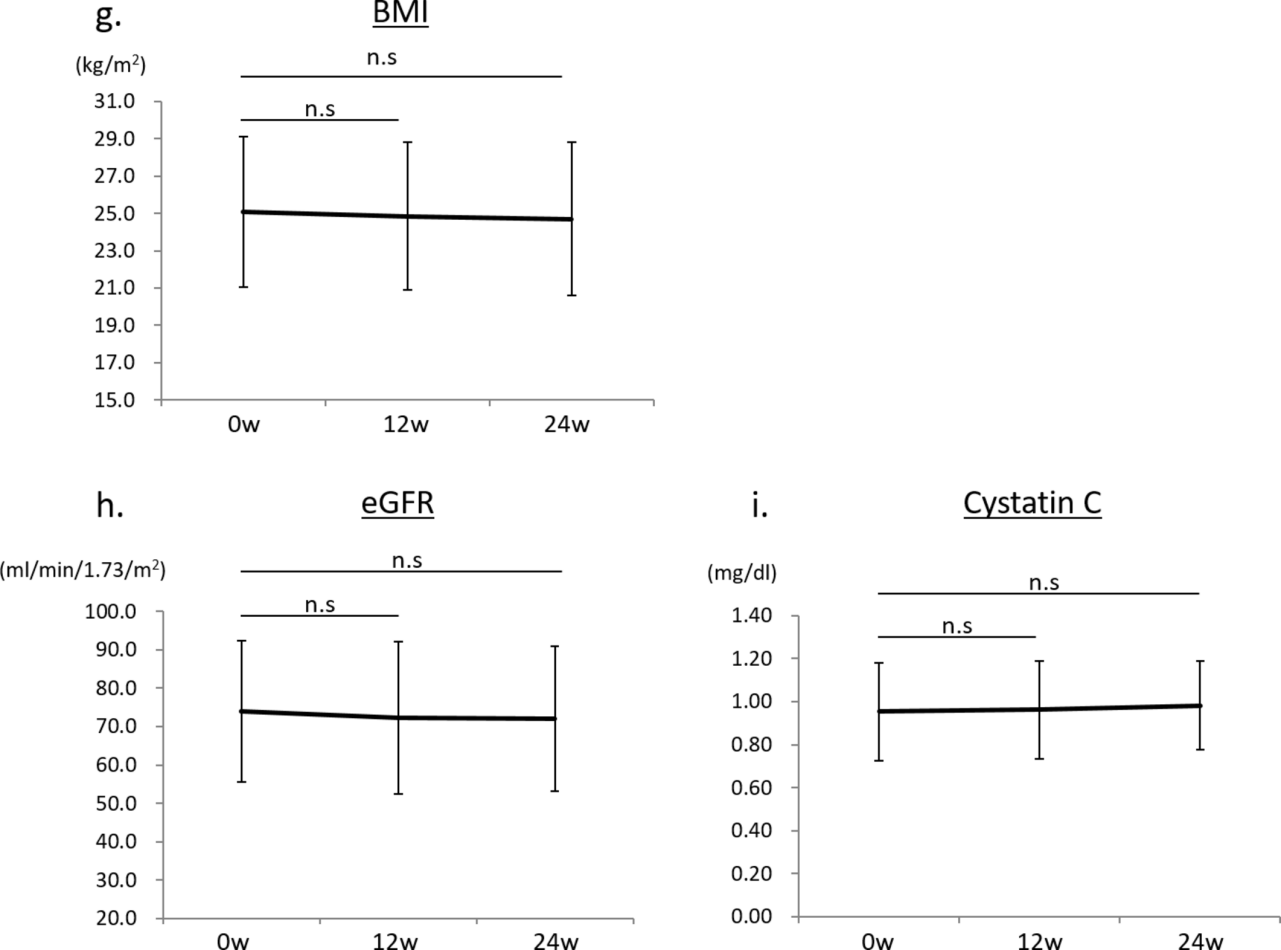
(G) Body mass index (BMI) values at baseline and after treatment with anagliptin in 20 participants at 12 and 24 weeks. (H) Estimated glomerular filtration rate (eGFR) at baseline and after treatment with anagliptin in 20 participants at 12 and 24 weeks. (I) Serum cystatin C values at baseline and after treatment with anagliptin in 20 participants at 12 and 24 weeks. Error bars represent SD. n.s denotes not significant.

**Figure 4:**
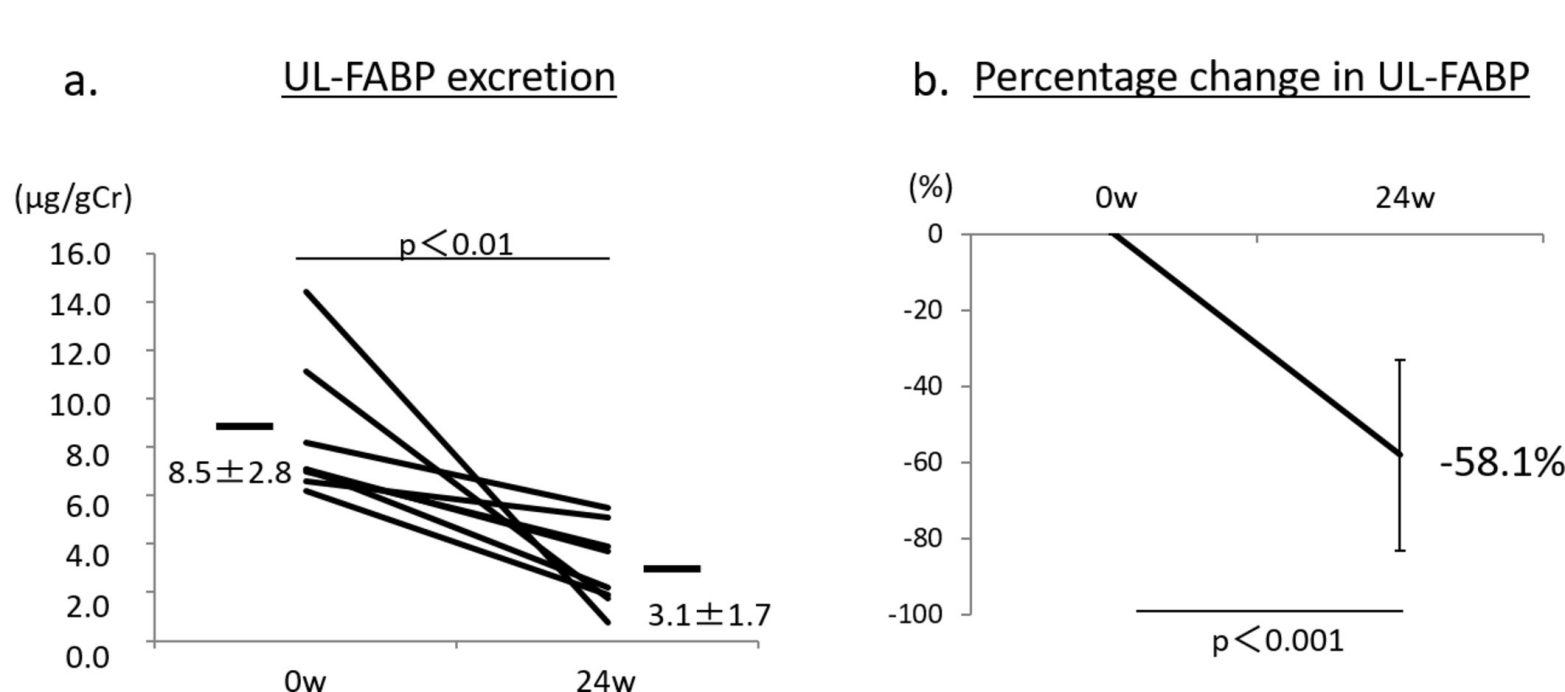
(A) Urinary L-FABP (ULFABP) values in eight participants who had more than 5 µg/g creatinine (Cr) at the start of the study, at baseline and after treatment with anagliptin at 24 weeks. p<0.01 versus baseline. (B) Percentage change in UFABP (Δ%UFABP) in eight participants from baseline to after treatment with anagliptin at 24 weeks. p<0.001 versus baseline. Error bars represent SD. L-FABP, liver-type  fatty acid-binding protein.

The single linear regression analysis between Δ%UACR and age, duration of diabetes, ΔHbA1c, ΔLDL-C, Δsystolic BP or ΔBMI for 24 weeks, and HbA1c, UACR, BMI and eGFR at baseline, did not show significant correlation (table 3). In addition, there was no relationship between Δ%ULFABP and ΔHbA1c after 24 weeks, which was evaluated by a single linear regression analysis (r=0.547, 95% CI −0.270 to 1.48, p=0.165).

**Table 3:** The single linear regression analysis between Δ%UACR and clinical parameters

	r	95% CI	p Value
Δ% UACR			
Age (year)	−0.180	−0.293 to 0.658	0.447
Duration of diabetes (year)	0.317	−0.147 to 0.804	0.173
ΔHbA1c (%)	0.039	−0.437 to 0.514	0.871
ΔSystolic BP (mm Hg)	−0.292	−0.190 to 0.790	0.226
ΔBMI (kg/m^2^)	−0.002	−0.473 to 0.477	0.993
ΔLDL-C (mg/dL)	−0.149	−0.325 to 0.625	0.531
ΔTG (mg/dL)	−0.250	−0.220 to 0.730	0.288
ΔHDL-C (mg/dL)	0.033	−0.442 to 0.509	0.890
ΔeGFR (mL/min/1.73 m^2^)	0.180	−0.294 to 0.657	0.448
HbA1c at baseline (%)	−0.219	−0.253 to 0.698	0.354
UACR (log) at baseline (mg/g Cr)	−0.010	−0.465 to 0.486	0.965
BMI at baseline (kg/m^2^)	−0.323	−0.140 to 0.811	0.164
eGFR at baseline (mL/min/1.73 m^2^)	−0.305	−0.161 to 0.790	0.191

Data are the results of a single linear regression analysis for variables at 24 weeks.

BP, blood pressure; BMI, body mass index; eGFR, estimated glomerular filtration rate; HbA1c, hemoglobin A1c; HDL-C, high-density lipoprotein-cholesterol; LDL-C, low-density lipoprotein-cholesterol; TG, triglyceride; UACR, urinary albumin to creatinine ratio.

## Discussion

The present study showed that the administration of anagliptin for 24 weeks significantly decreased the UACR from the baseline in a glucose-independent, lipid-independent and BP-independent manner. In addition, treatment with anagliptin significantly reduced the levels of urinary L-FABP excretion in the participants, who had more than 5 µg/g Cr at baseline, independent of the change in HbA1c. This is the first report showing a renoprotective effect of anagliptin on patients with type 2 diabetes with nephropathy.

DPP-4 is an enzyme that cleaves and inactivates incretin hormones capable of stimulating insulin secretion from pancreatic β cells. DPP-4 inhibitors are now widely used for the treatment of type 2 diabetes. Previous reports have shown that currently available DPP-4 inhibitors, including anagliptin, exert a glucose-lowering effect in patients with diabetes,^29^ and there is no significant difference in their glucose-lowering efficacy. Our data also demonstrated that HbA1c was significantly decreased at 12 weeks and 24 weeks from baseline levels by additional treatment with anagliptin. However, switching to anagliptin from other DPP-4 inhibitors such as sitagliptin, alogliptin, vildagliptin, linagliptin or teneligliptin exhibited no significant change in HbA1c levels after 12 and 24 weeks of treatment with anagliptin in 20 participants. In addition to the glucose-lowering effect, previous reports have shown that anagliptin has a lipid-lowering effect, decreasing the plasma total cholesterol, LDL-C and TG levels, which was indicated by pooled analysis of phase III clinical trials.^30^ However, in this study, administration of anagliptin showed no change in lipid data after both 12 and 24 weeks of treatment. Although 70% of participants received lipid-lowering drugs, including statins, fibrate or ezetimibe, anagliptin did not show lipid-lowering effects, independent of receiving lipid-lowering drugs or not receiving them. It is unclear why anagliptin exhibited no lipid-lowering effects in this study.

Previous clinical studies have shown a beneficial effect of DPP-4 inhibitors in diabetic nephropathy. Sitagliptin reduced albuminuria in several uncontrolled trials and a small randomized controlled trial, and the reduction of albuminuria was independent of the decrease in HbA1c,^14 17^ and correlated with decreases in both systolic BP and eGFR.^31^ Groop *et al*,^23^ in a pooled analysis of four studies, demonstrated that treatment with linagliptin in addition to RAS inhibitors reduced UACR by 32% and by 6% compared with placebo, and the efficacy of linagliptin was unaffected by baseline HbA1c levels or systolic BP, changing of HbA1c or systolic BP during the treatment with linagliptin. Therefore, the effect of linagliptin on the reduction of albuminuria was exerted in a glucose-independent and BP-independent manner. Tani *et al* 26 also evaluated the effects of vildagliptin on atherogenic LDL-C heterogeneity and albuminuria in subjects with diabetes. The UACR decreased significantly by ∼45% after 8 weeks of treatment with vildagliptin, and reduction of UACR by vildagliptin was correlated with change in HbA1c, lipid data, systolic BP and eGFR. Thus, clinically, DPP-4 inhibitors may improve albuminuria and may have a renoprotective effect; however, further study is necessary to identify whether long-term treatment with several DPP-4 inhibitors in patients with diabetes may maintain renal function as well as reduction of albuminuria.

There are few reports regarding comparative data among DPP-4 inhibitors on the renoprotective effect, including reduction of albuminuria. Fujita *et al* 24 reported that in a crossover study with two DPP-4 inhibitors, sitagliptin and alogliptin, in patients with type 2 diabetes who have microalbuminuria and take ARBs, switching from sitagliptin to alogliptin reduced UACR in a glucose-lowering-independent manner. Switching to alogliptin from sitagliptin significantly reduced urinary 8-hydroxy-2’-deoxyguanosine (8-OHdG) excretion, and increased the plasma levels of stromal cell-derived factor-1α (SDF-1α), which is one of the substrates of DPP-4. Therefore, alogliptin might be more effective in the reduction of albuminuria compared with sitagliptin. However, the detailed mechanism is still unclear. In our study, the Δ%UACR from baseline to 12 or 24 weeks after anagliptin treatment was significantly lower, in a glucose-independent, lipid-independent, BP-independent or use of RAS inhibitors-independent manner. The reduction in the UACR induced by anagliptin might be observed by switching from sitagliptin, alogliptin and teneligliptin. By contrast, switching from vildagliptin and linagliptin to anagliptin seemed to show no effect on urinary albumin excretion, but the sample number was very small. What is the difference in the effect of reduction of the UACR among each of the DPP-4 inhibitors? Previously, we reported that linagliptin, but not sitagliptin, inhibits the homodimer formation of DPP-4, which is related to DPP-4 activation, in cultured endothelial cells,^32^ and this difference may be one of the reasons why the two drugs display different properties. DPP-4 is widely expressed in many cell types, including renal endothelial cells and epithelial tubular cells; therefore, different DPP-4 inhibitors may exhibit diverse biological influences depending on the cell type. However, it is unclear whether anagliptin can inhibit the homodimer formation of DPP-4, similar to linagliptin. In addition, differences in the binding modes in the active site of DPP-4 and the form of binding may contribute to the different effects exhibited by DPP-4 inhibitors. A previous report analyzing the single-crystal structure and enzyme interactions showed that the interacting subsites of anagliptin with DPP-4 are the S1, S2 and S2 extensive subsites, and anagliptin is included in class 3 according to the categorization by Nabeno *et al*.^33^ This binding mode of anagliptin leads to high and selective DPP-4 inhibition. Furthermore, anagliptin binds to Ser630 of DPP-4, which is a catalytic residue and a center of its activation in the S1 subsite, through a dipole interaction of the cyanopyrrolidine structure, and anagliptin may possibly lead to the formation of the imidate intermediates through covalent binding to DPP-4.^34^ Vildagliptin also binds to DPP-4 through covalent binding, which is thought to be a strong binding form.^33^ Therefore, inhibition of DPP-4 activity due to strength of binding to DPP-4 may be related to the renoprotective effect of anagliptin. In addition, anagliptin is taken twice a day, and therefore the peak of inhibition of DPP-4 activity occurs twice a day, which can lead to strong suppression of DPP-4 activity in the kidney, compared with other DPP-4 inhibitors such as sitagliptin, alogliptin and teneligliptin, which are taken once a day. Uchino and Kaku^35^ reported that administration of anagliptin twice a day exhibited the significantly increased plasma levels of active GLP-1, particularly after dinner, compared with those in the treatment with sitagliptin once a day, in an open-label, two-period crossover study. Thus, differences in the chemical structure, binding mode of DPP-4 inhibitors and the number of the peak of inhibition of DPP-4 activity may cause different effects on renoprotection; however, further studies are necessary to elucidate differences between anagliptin and other DPP-4 inhibitors, or whether anagliptin has a better effect than other DPP-4 inhibitors, particularly sitagliptin, alogliptin and teneligliptin, on renoprotection.

Urinary L-FABP is one of the markers for tubulointerstitial damage and an oxidative stress marker. Araki *et al* reported that urinary L-FABP of more than 5 µg/g Cr may be a predictive marker for renal and cardiovascular prognosis in patients with type 2 diabetes without advanced nephropathy.^7 8^ Therefore, we evaluated the effect of anagliptin on urinary excretion in patients who had a urinary L-FABP level of more than 5 µg/g Cr. Interestingly, anagliptin clearly decreased the excretion of urinary L-FABP, which indicates a reduction of tubulointerstitial damage, tubular hypoxia and oxidative stress. There are no reports showing a beneficial effect of DPP-4 inhibitors on urinary L-FABP excretion. However, since we could not measure the oxidative stress marker such as urinary 8-OHdG excretion, it is unclear whether anagliptin may provide renal protective effect via stronger antioxidative action than other DPP-4 inhibitors. Thus, our data indicate that anagliptin may suppress both albuminuria and urinary L-FABP, which are predictive markers for renal and cardiovascular prognosis, indicating improvement of glomerular/tubulointerstitial damage, possibly inhibiting the progression of diabetic nephropathy and CVD.

Experimental studies have suggested a renoprotective role of DPP-4 inhibitors in various models of chronic kidney disease (CKD), including diabetic nephropathy, which may be independent of lowering glucose levels. The renoprotective effect of DPP-4 inhibitors in diabetic nephropathy may be exerted through an increase in active GLP-1 or through the inhibition of DPP-4 itself. Previous reports show that GLP-1 receptor agonists may prevent disease progression in diabetic nephropathy through direct effects on the GLP-1 receptor in renal cells including glomerular endothelial cells and monocytes/macrophages.^36 37^ Higashijima *et al* 38 also demonstrated that DPP-4 inhibitors, including anagliptin, reduced macrophage infiltration directly via GLP-1-dependent signaling in a rat Thy-1 nephritis model. Therefore, increased GLP-1 induced by DPP-4 inhibition may also lead to renal protection through the GLP-1 receptor and its signaling.^39^ By contrast, several reports showed that the inhibition of DPP-4 ameliorates kidney injury animal models, including diabetic nephropathy. Tanaka *et al* 40 also demonstrated that linagliptin significantly inhibited tubulointerstitial injury induced by peritoneal injection of free fatty acid-bound albumin, such as inflammation, fibrosis and apoptosis, in mice without altering blood glucose levels. The anti-inflammatory effect of DPP-4 inhibition in monocytes/macrophages is also associated with renoprotection. In an apolipoprotein E-deficient atherosclerotic mice model, not a kidney disease model, Ervinna *et al* 41 demonstrated that anagliptin exerted an antiatherosclerotic effect through inhibition of the inflammatory reaction of monocytes and inhibition of smooth muscle cell proliferation. Shinjo *et al* 42 also demonstrated that anagliptin attenuated inflammatory cytokine expression in lipopolysaccharide-stimulated macrophage, adipocytes and hepatocytes. The in vitro suppressive effects on cytokine production in cultured macrophages by anagliptin suggest the anti-inflammatory effects of these DPP-4 inhibitors to be direct actions rather than via increased concentrations of incretins such as GLP-1. Furthermore, they showed that sitagliptin also exerted anti-inflammation, as well as that of anagliptin; however, the effect of sitagliptin is weaker than that of anagliptin. The treatment with anagliptin and sitagliptin resulted in similar inhibitory effects on DPP-4 activity in the supernatants of both cultured macrophages and adipocytes, whereas anagliptin more strongly inhibited DPP-4 activity in both cell lysates than sitagliptin. The difference in the degrees of anti-inflammatory effects between anagliptin and sitagliptin may be explained by different inhibitory efficiencies against DPP-4 in cell lysates (cell surface DPP-4) and supernatants (soluble form of DPP-4). Oxidative stress also plays a crucial role for the pathogenesis of diabetic nephropathy. Mega *et al* 43 showed that sitagliptin ameliorated diabetic nephropathy in Zucker diabetic fatty rat, accompanied by reduced lipid peroxidation. Furthermore, teneligliptin works as a direct scavenger of hydroxyl radicals, resulting in reduction of oxidative stress.^44^ There are few reports regarding the renoprotective effect of anagliptin in both experimental animal models and in human data. Therefore, further study is necessary to evaluate these points.

There were several limitations in our study design. It was a non-controlled observational study that occurred over a short time period, and the number of participants was small. DPP-4 cleaves a lot of substrates (peptides) including GLP-1, GIP, SDF-1α, brain natriuretic peptide (BNP) and so on. Although we could not show the changes in the levels of these peptides following anagliptin treatment, these changes may be involved in renal protection observed in this study. In addition, we could not evaluate oxidative stress or inflammation to assess the mechanism of the beneficial effect on the diabetic kidney. Therefore, the mechanism by which anagliptin reduced the UACR and urinary L-FABP excretion in a glucose-lowering independent manner is unclear. Further study is necessary to elucidate these points.

In conclusion, in the present study of just 24 weeks’ duration, anagliptin caused a decrease in the UACR and urinary L-FABP, which are prognostic markers for CKD and CVD, and the decrease was independent of any change in HbA1c. Therefore, anagliptin may have potential for halting the progression of diabetic nephropathy and the development of CVD through a renoprotective effect.

10.1136/bmjdrc-2017-000391.supp2
