# Supplementary figures and images for: Anagliptin ameliorates albuminuria and urinary liver-type fatty acid-binding protein excretion in patients with type 2 diabetes with nephropathy in a glucose-lowering-independent manner
Source: BMJ Open Diabetes Res Care. 2017 Jul 7;5(1):e000391. doi: 10.1136/bmjdrc-2017-000391 (PMC5530236; doi:10.1136/bmjdrc-2017-000391)

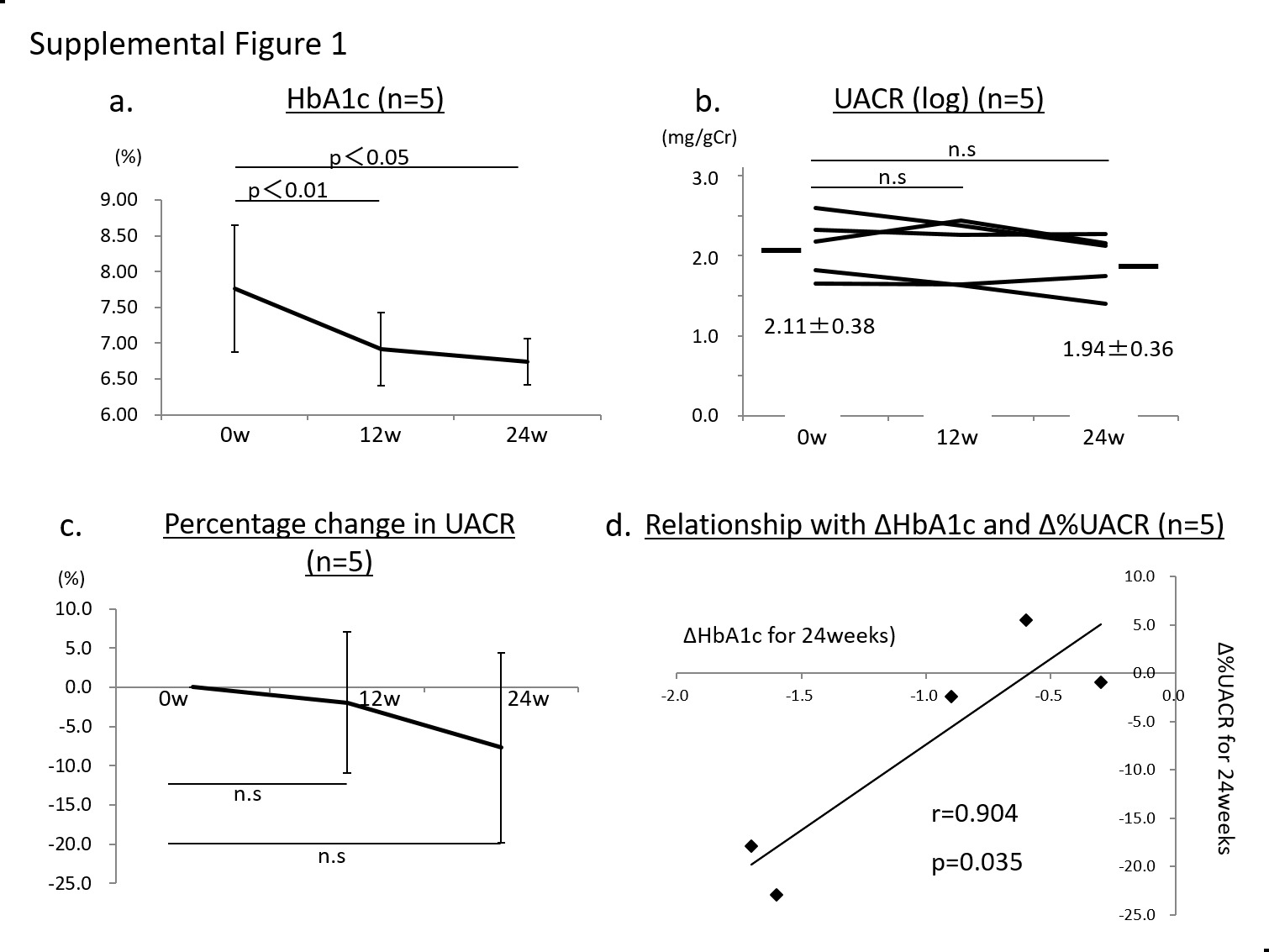

Supplement: Supplementary data 1 [file bmjdrc-2017-000391supp002.jpg]
