# Supplementary material for: Anagliptin ameliorates albuminuria and urinary liver-type fatty acid-binding protein excretion in patients with type 2 diabetes with nephropathy in a glucose-lowering-independent manner
Source: BMJ Open Diabetes Res Care. 2017 Jul 7;5(1):e000391. doi: 10.1136/bmjdrc-2017-000391 (PMC5530236; doi:10.1136/bmjdrc-2017-000391)
Supplement: Supplementary file 3 [file bmjdrc-2017-000391supp003.htm]

**Supplemental Figure Legend**

Figure 1.

a: HbA1c
values at baseline and after treatment with additional anagliptin in 5
participants at 12 and 24 weeks. p < 0.01 and p < 0.05 versus baseline.
Error bars represent standard deviation (SD).

b: Urinary albumin/Cr ratio
(UACR) (log)
values in 5 participants at baseline and after treatment with anagliptin at 12
and 24 weeks. n.s
denotes not significant.

c: Percentage
change in the urinary
albumin/Cr ratio (UACR) from baseline to after treatment with anagliptin in 5
participants at 12 and 24 weeks. Error bars represent SD. n.s denotes not
significant.

d:
Relationship between ΔHbA1c and Δ%UACR at 24 weeks after treatment with anagliptin in 5 participants. (r =
0.904, p = 0.035)
